# Supplementary material for: Histone Deacetylase 3 Governs β-Estradiol-ERα-Involved Endometrial Tumorigenesis via Inhibition of STING Transcription
Source: Cancers (Basel). 2022 Sep 28;14(19):4718. doi: 10.3390/cancers14194718 (PMC9563443; doi:10.3390/cancers14194718)
Supplement: Supplementary file 1 [file cancers-14-04718-s001.zip › cancers-1886717-File S1.pptx]

## Slide 1
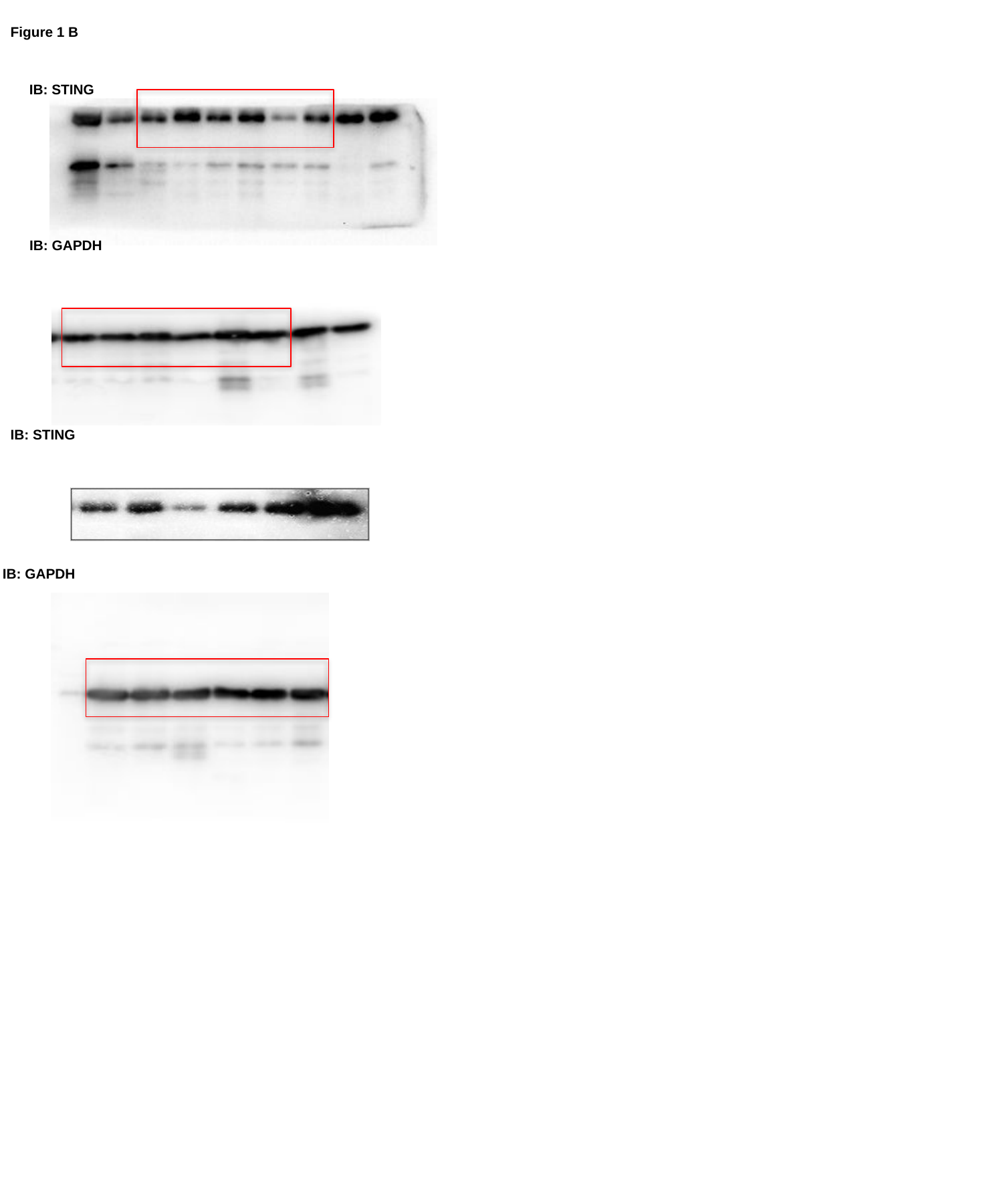

Figure 1 B
IB: STING
IB: GAPDH
IB: STING
IB: GAPDH

## Slide 2
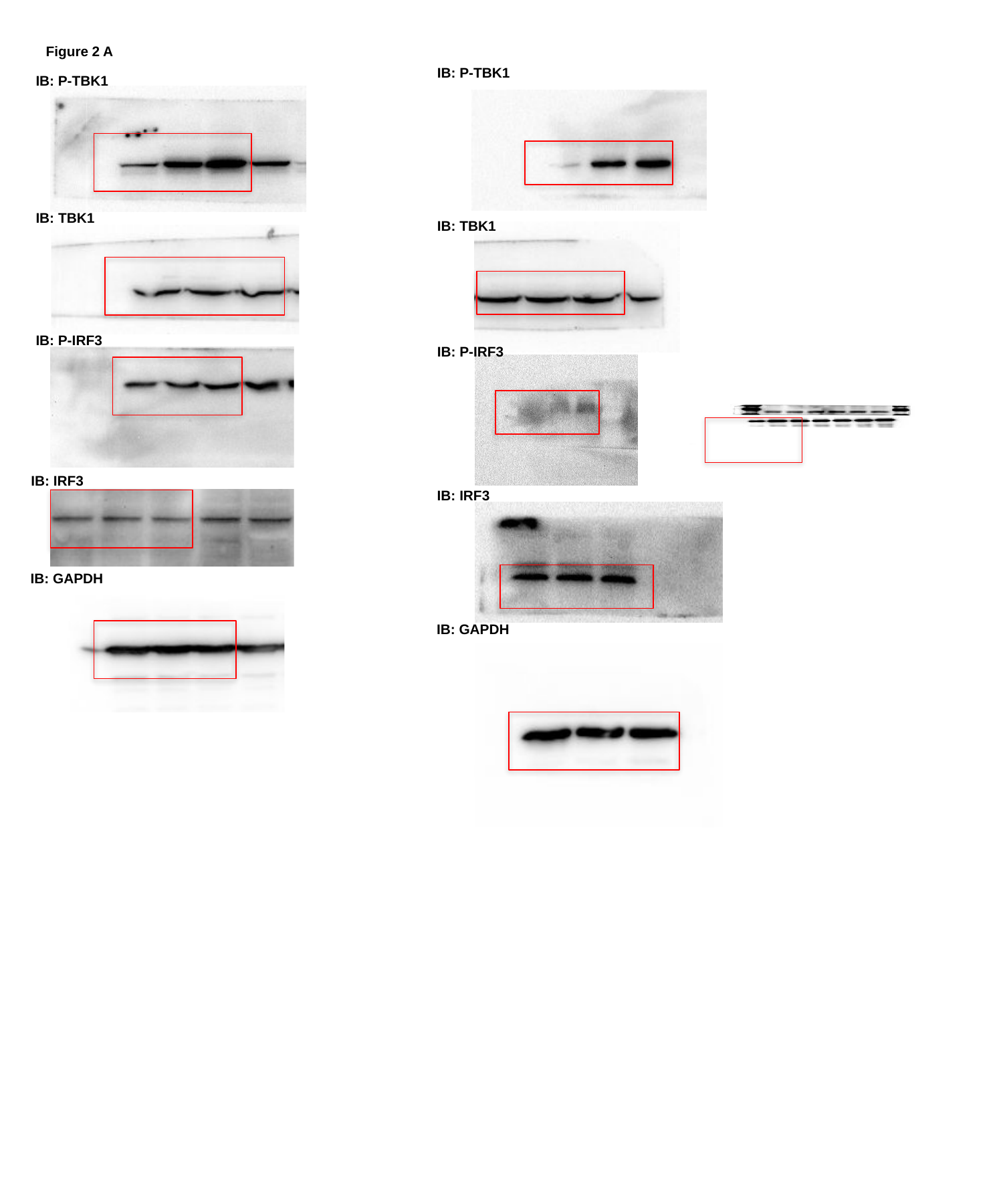

Figure 2 A
IB: P-TBK1
IB: P-TBK1
IB: TBK1
IB: TBK1
IB: P-IRF3
IB: P-IRF3
IB: IRF3
IB: IRF3
IB: GAPDH
IB: GAPDH

## Slide 3
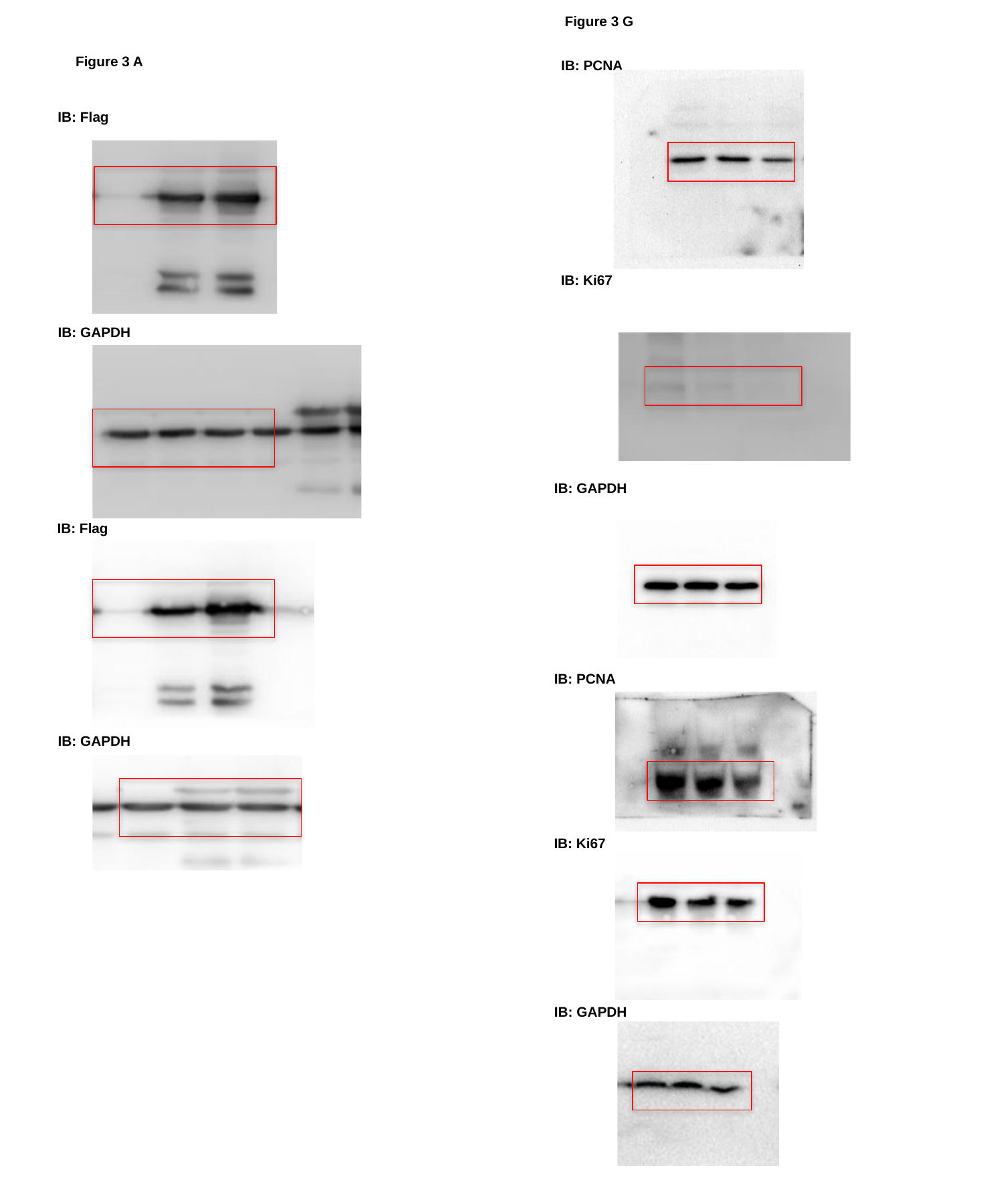

Figure 3 G
Figure 3 A
IB: PCNA
IB: Flag
IB: Ki67
IB: GAPDH
IB: GAPDH
IB: Flag
IB: PCNA
IB: GAPDH
IB: Ki67
IB: GAPDH

## Slide 4
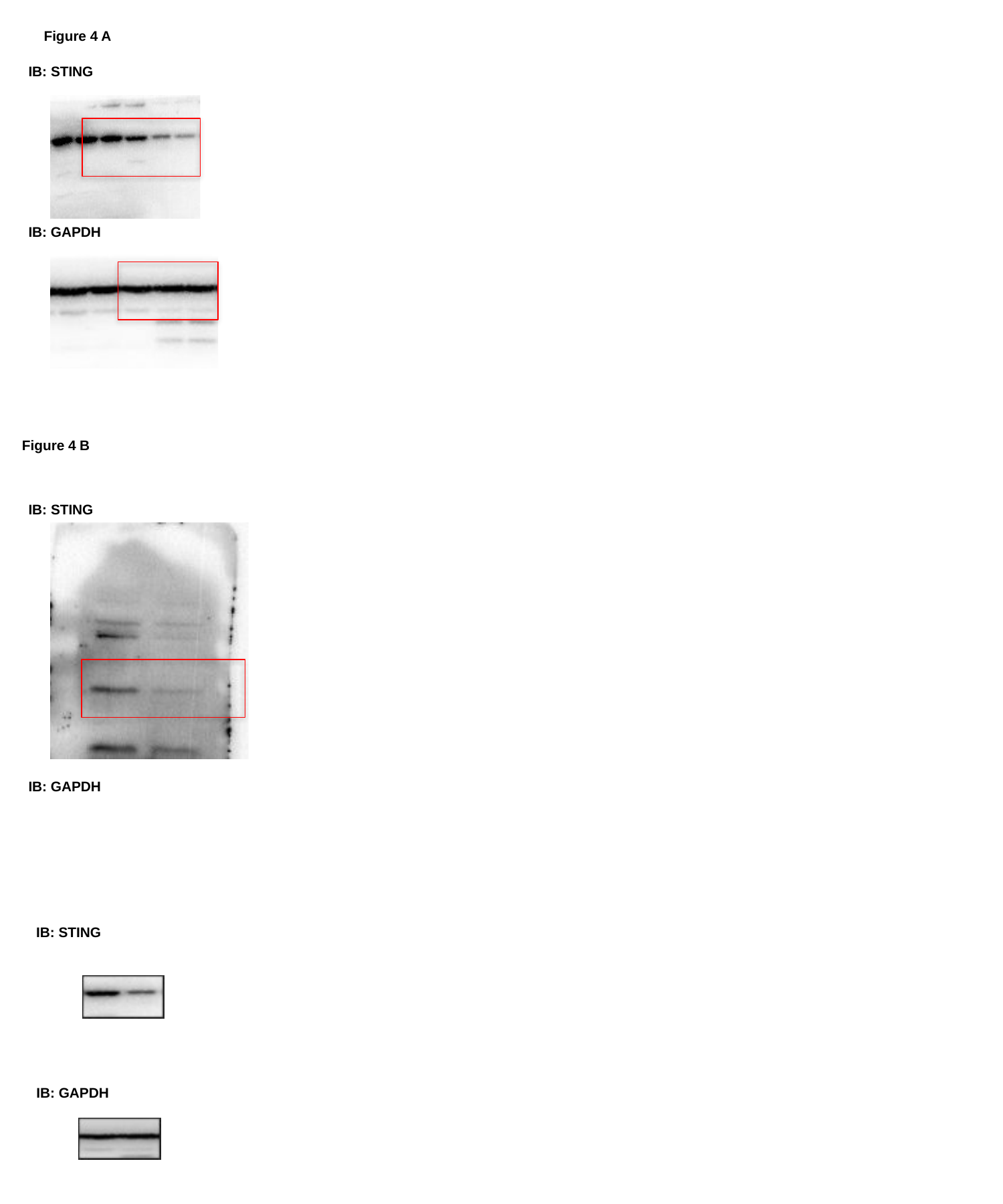

Figure 4 A
IB: STING
IB: GAPDH
Figure 4 B
IB: STING
IB: GAPDH
IB: STING
IB: GAPDH

## Slide 5
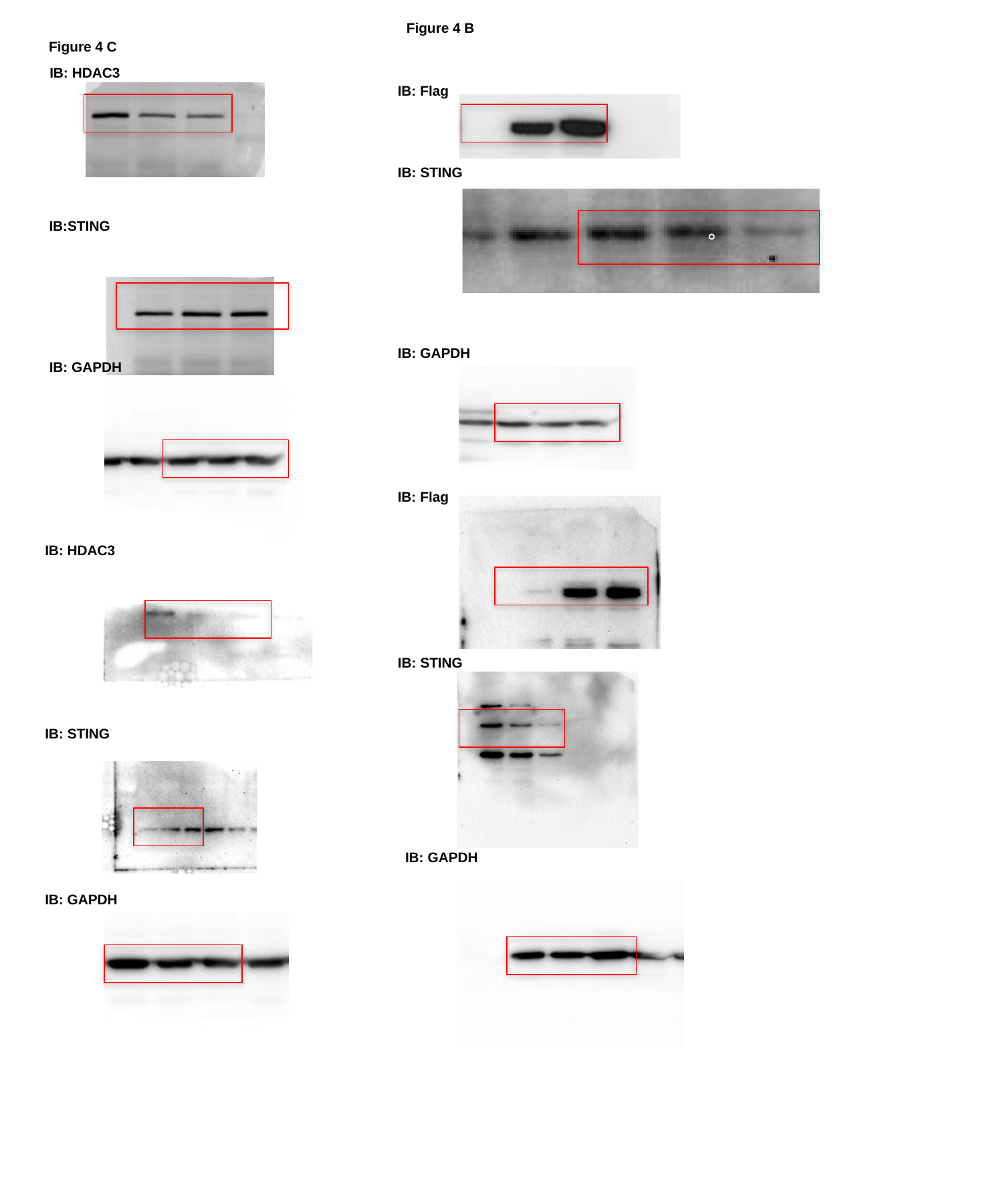

Figure 4 B
Figure 4 C
IB: HDAC3
IB: Flag
IB: STING
 。
IB:STING
IB: GAPDH
IB: GAPDH
IB: Flag
IB: HDAC3
IB: STING
IB: STING
IB: GAPDH
IB: GAPDH

## Slide 6
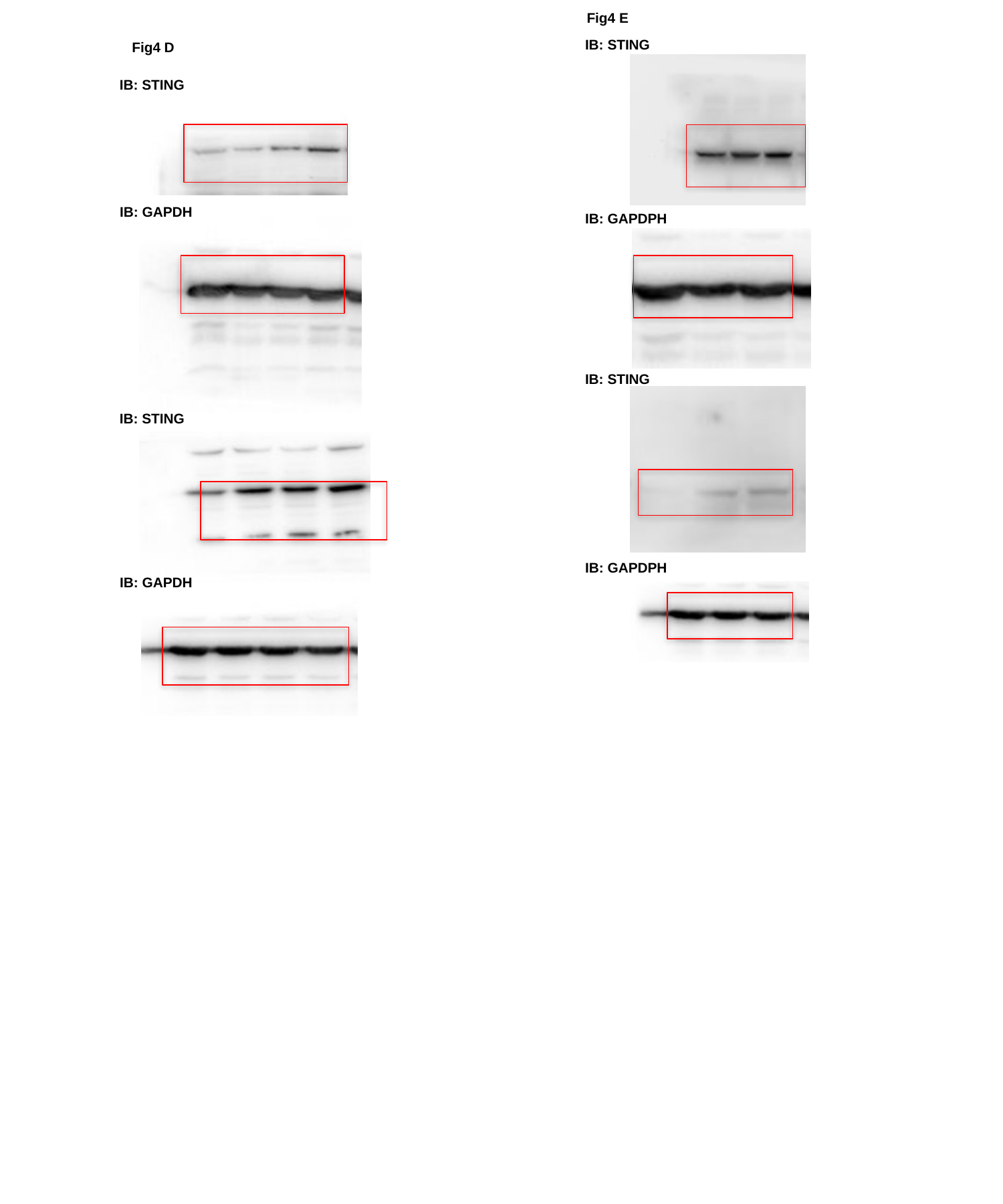

Fig4 E
IB: STING
Fig4 D
IB: STING
IB: GAPDH
IB: GAPDPH
IB: STING
IB: STING
IB: GAPDPH
IB: GAPDH

## Slide 7
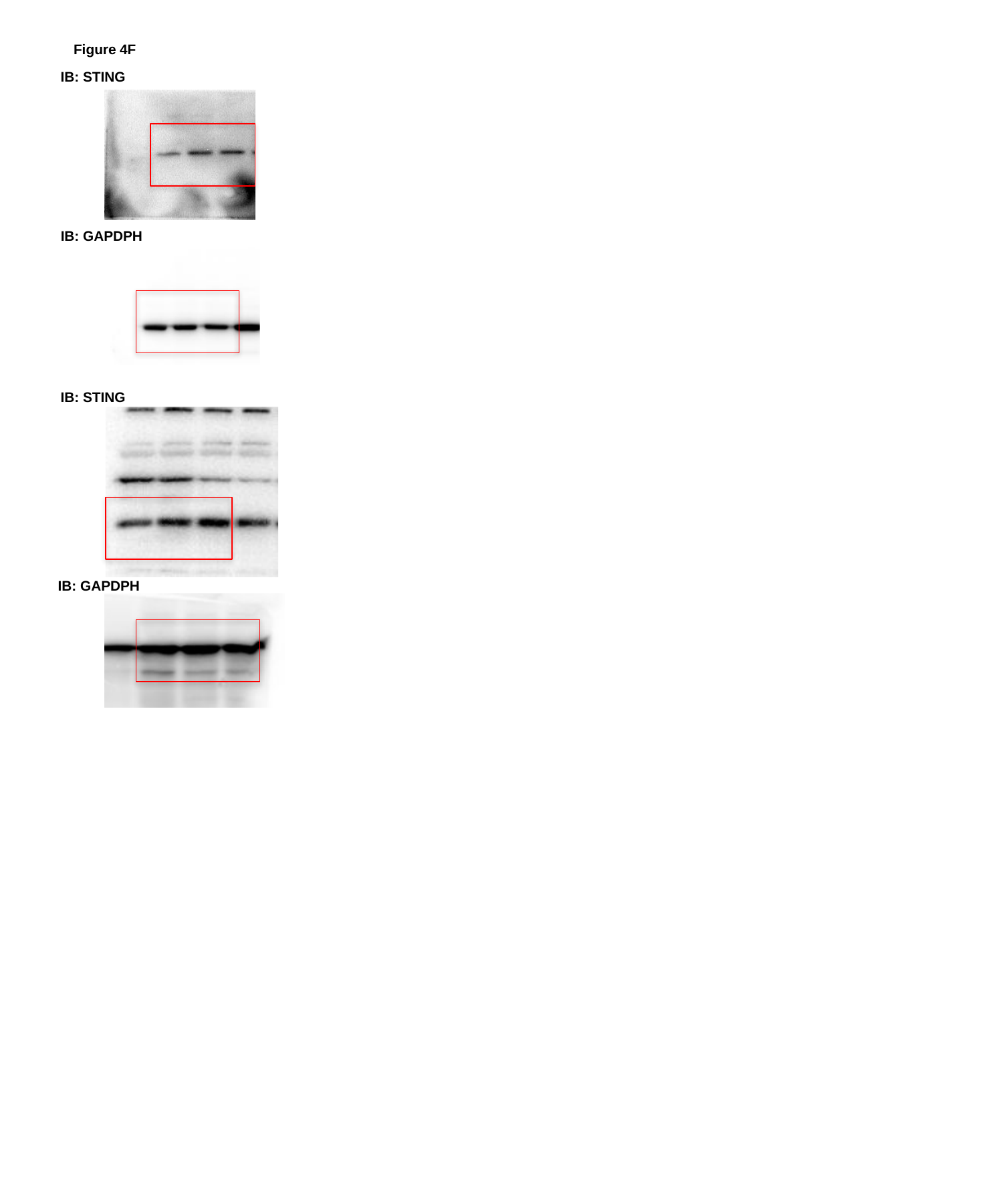

Figure 4F
IB: STING
IB: GAPDPH
IB: STING
IB: GAPDPH

## Slide 8
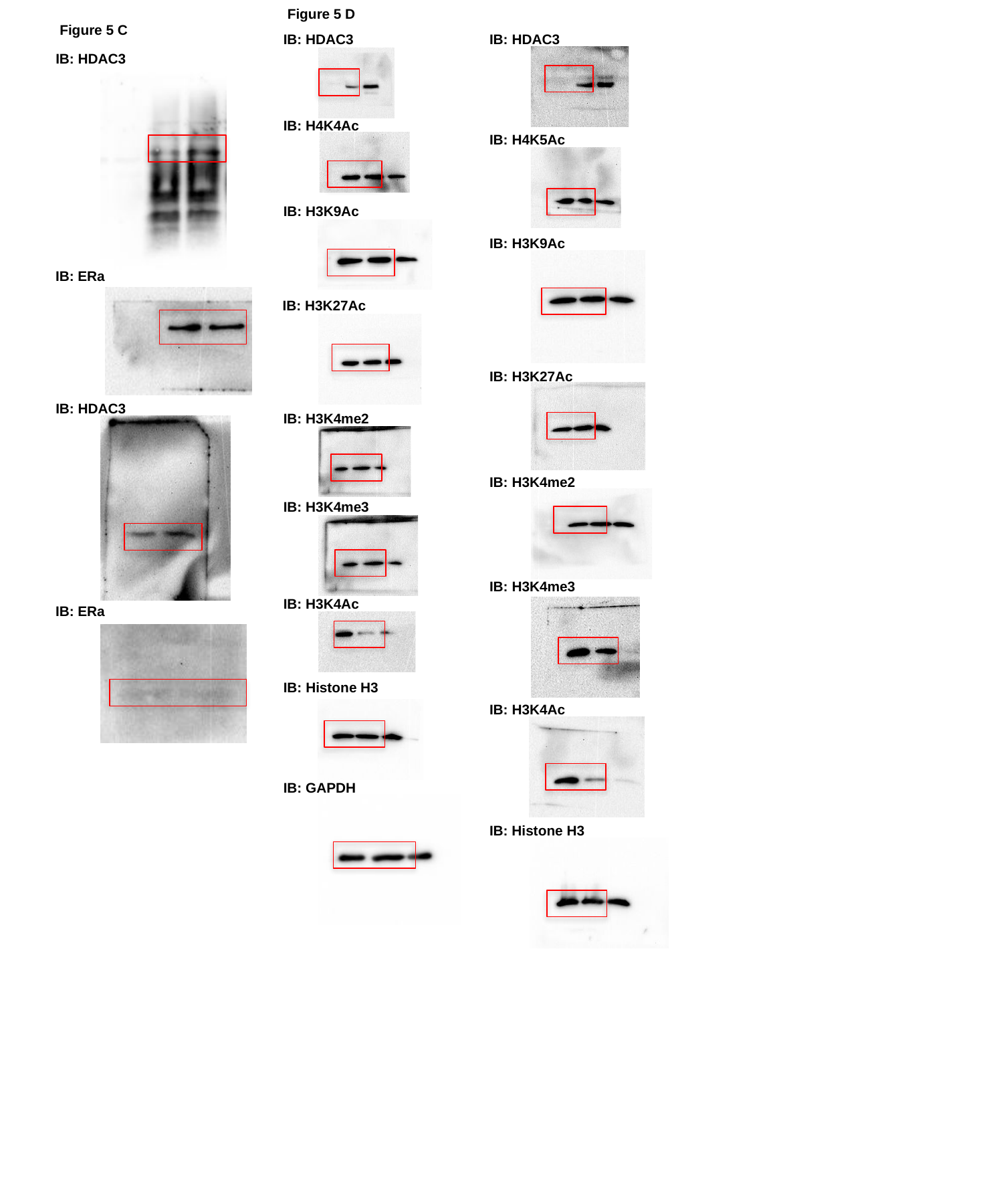

Figure 5 D
Figure 5 C
IB: HDAC3
IB: HDAC3
IB: HDAC3
IB: H4K4Ac
IB: H4K5Ac
IB: H3K9Ac
IB: H3K9Ac
IB: ERa
IB: H3K27Ac
IB: H3K27Ac
IB: HDAC3
IB: H3K4me2
IB: H3K4me2
IB: H3K4me3
IB: H3K4me3
IB: H3K4Ac
IB: ERa
IB: Histone H3
IB: H3K4Ac
IB: GAPDH
IB: Histone H3

## Slide 9
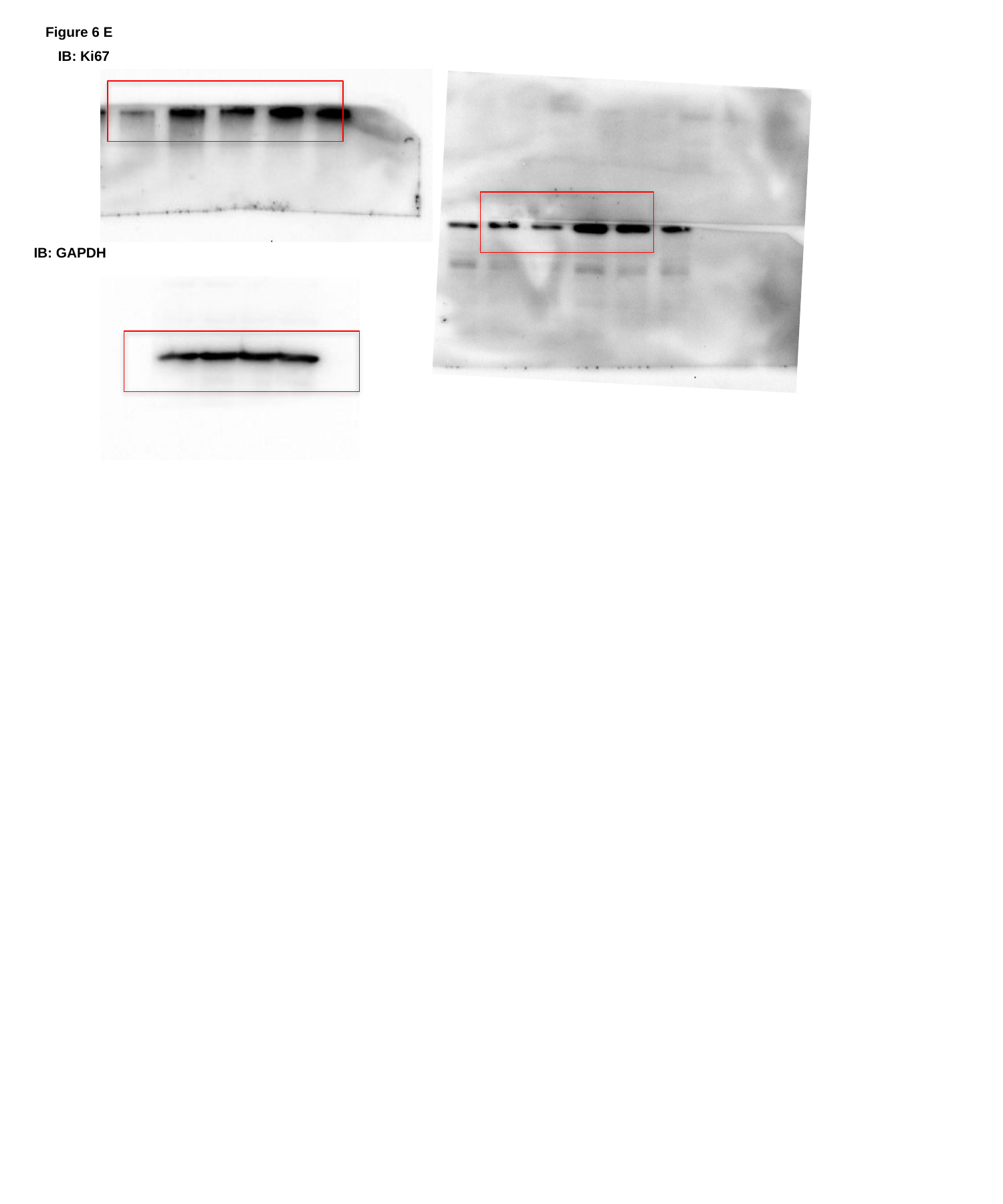

Figure 6 E
IB: Ki67
IB: GAPDH

## Slide 10
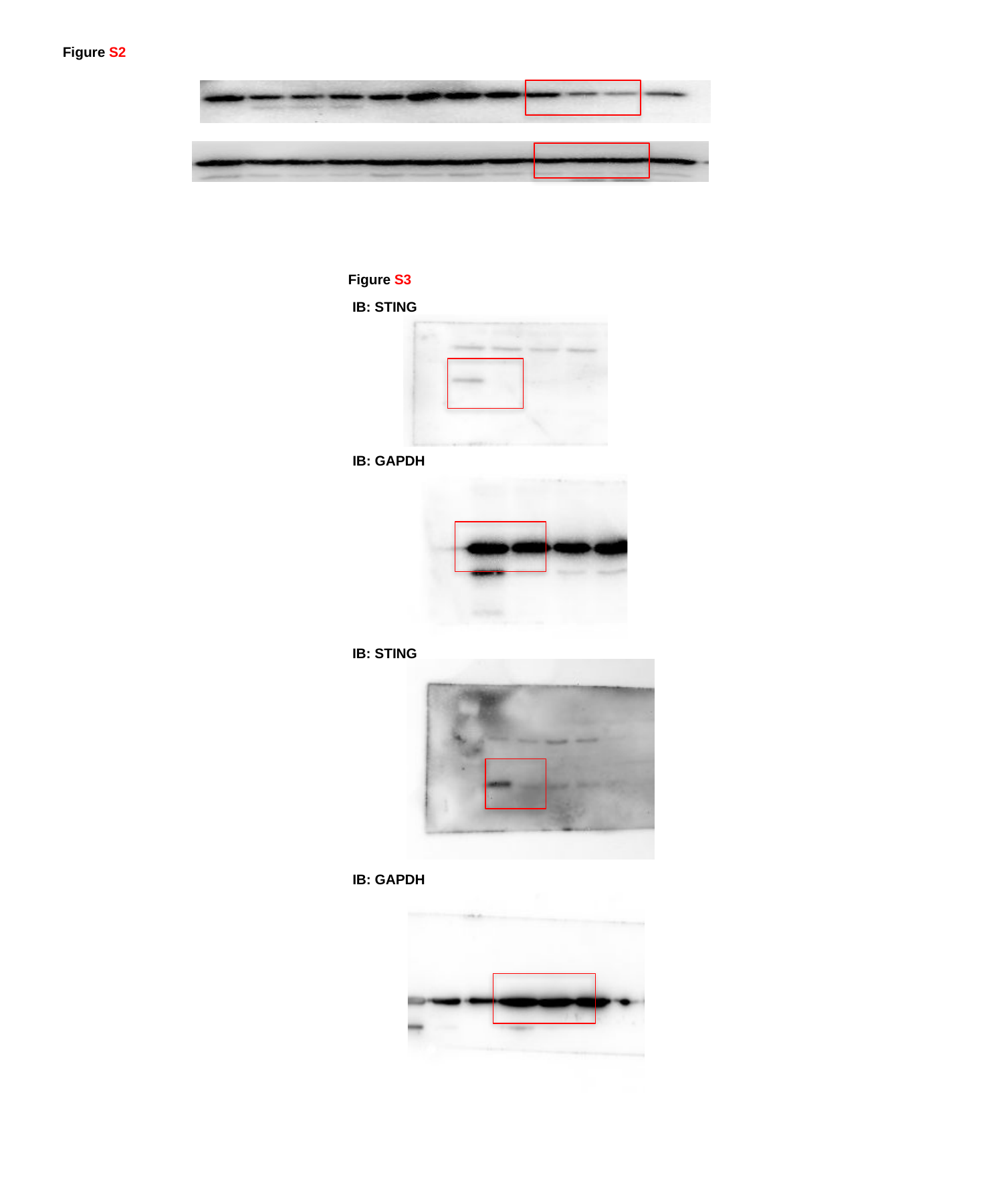

Figure S2
Figure S3
IB: STING
IB: GAPDH
IB: STING
IB: GAPDH

## Slide 11
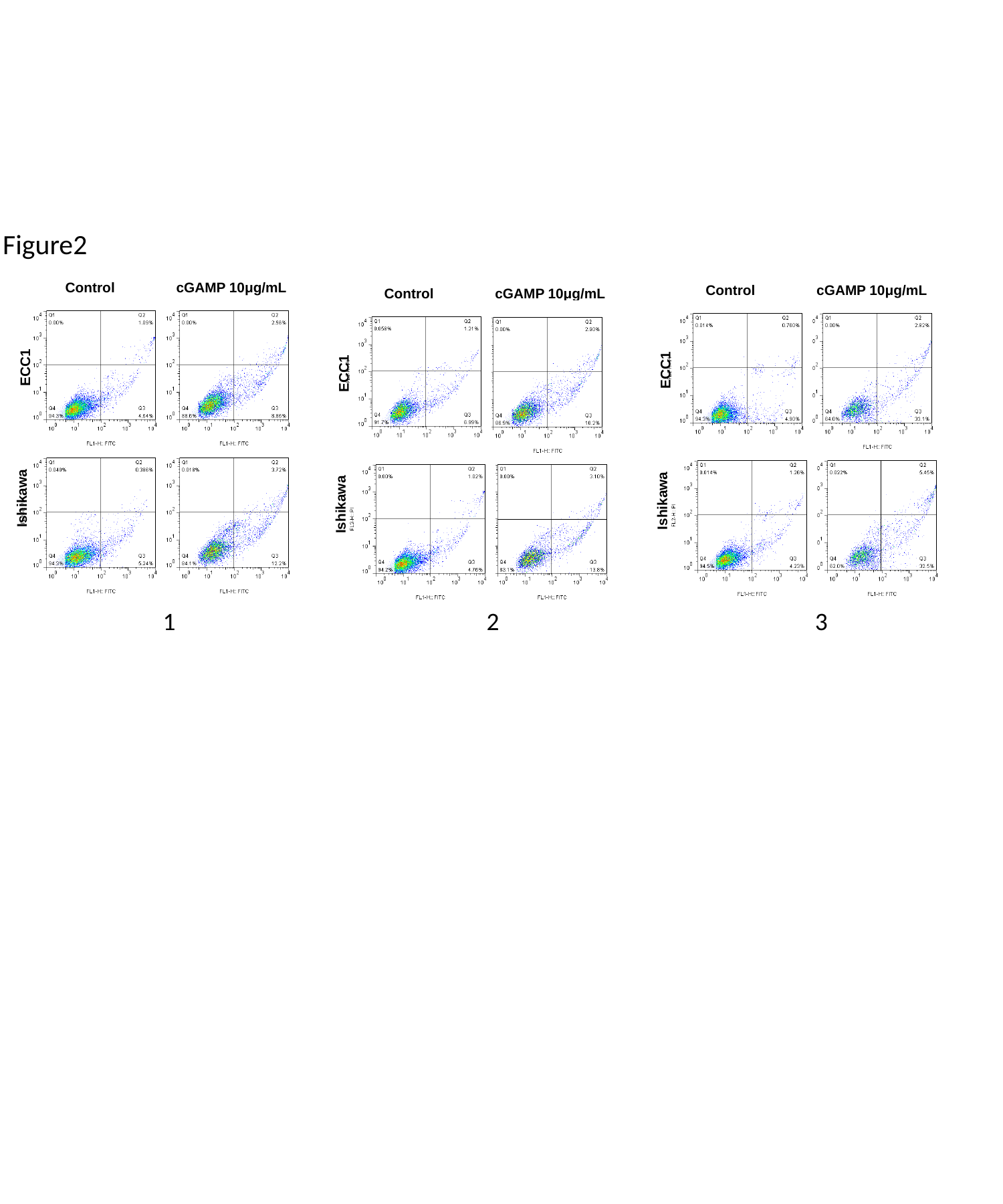

Figure2
 Control cGAMP 10μg/mL
ECC1
Ishikawa
 Control cGAMP 10μg/mL
 Control cGAMP 10μg/mL
ECC1
ECC1
Ishikawa
Ishikawa
1
2
3

## Slide 12
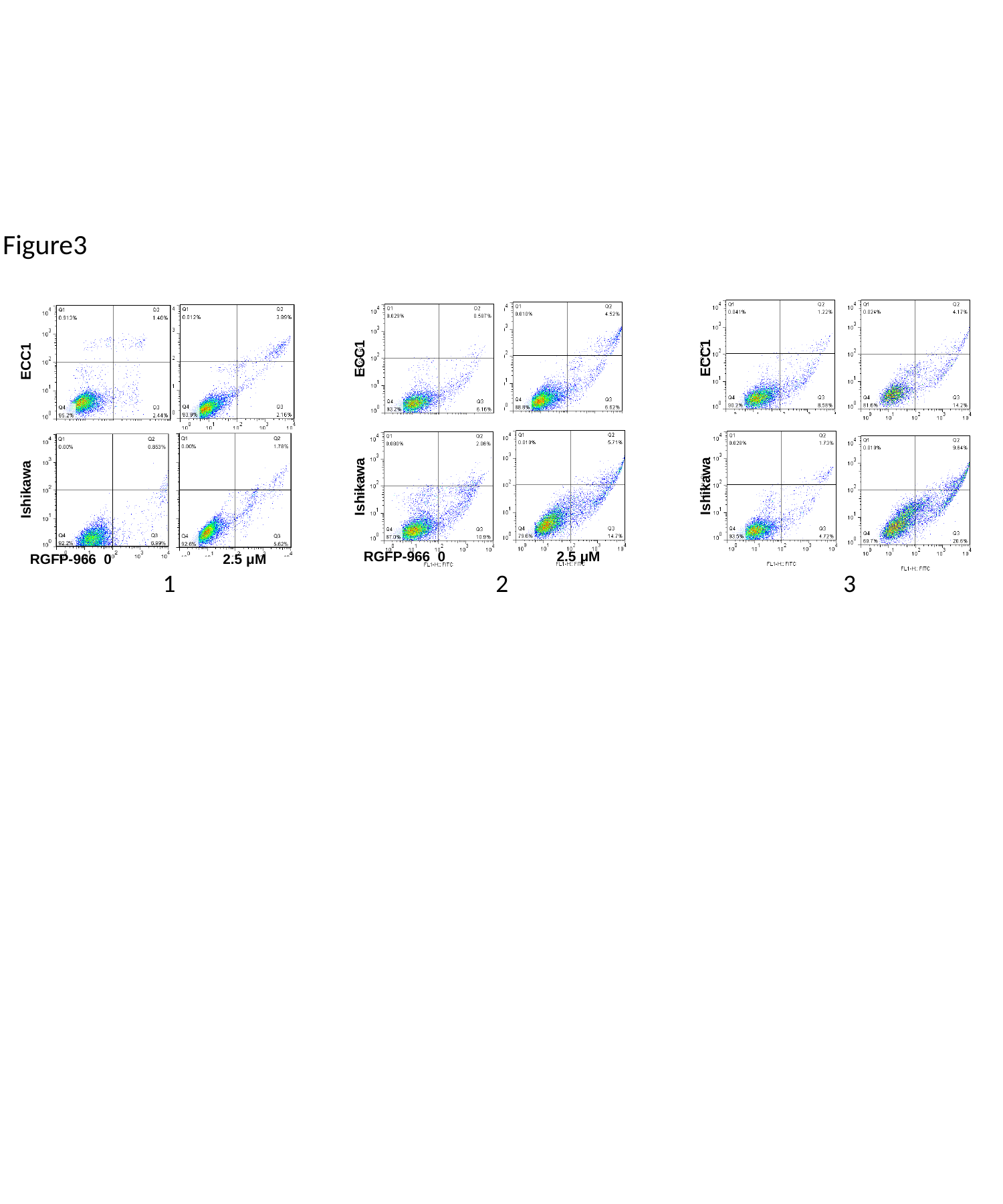

Figure3
ECC1
Ishikawa
RGFP-966 0 2.5 μM
ECC1
ECC1
Ishikawa
Ishikawa
RGFP-966 0 2.5 μM
1
2
3
RGFP-966 0 2.5 μM
