# Supplementary material for: Histone Deacetylase 3 Governs β-Estradiol-ERα-Involved Endometrial Tumorigenesis via Inhibition of STING Transcription
Source: Cancers (Basel). 2022 Sep 28;14(19):4718. doi: 10.3390/cancers14194718 (PMC9563443; doi:10.3390/cancers14194718)
Supplement: Supplementary file 1 [file cancers-14-04718-s001.zip › cancers-1886717-supplementary.pdf]

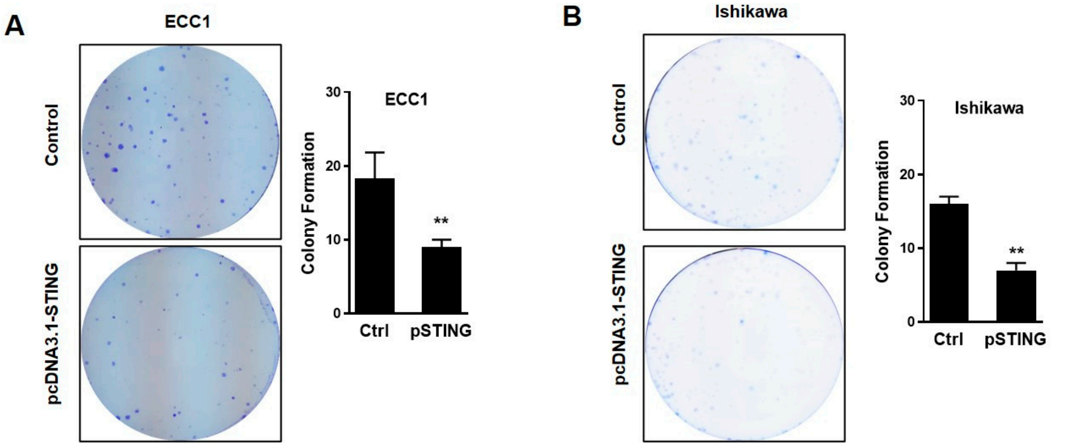

Figure S1. A-B) Colony formation assays of ECC1 (A) and Ishikawa cells (B) transfected with pcDNA3.1-STING construct.

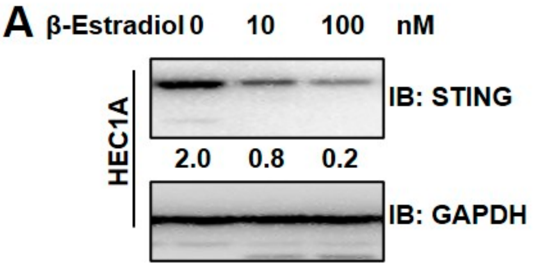

Figure S2. A) IB analysis of WCL derived from HEC1A cells.

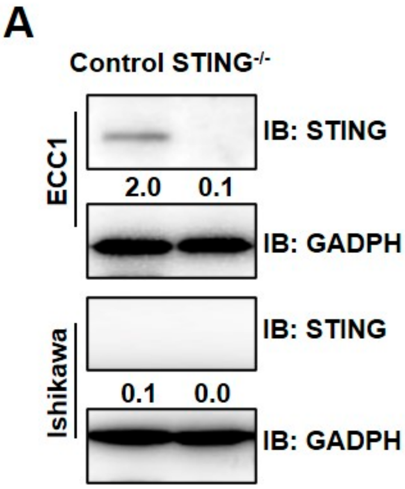

Figure S3. (A) IB analysis of WCL derived from ECC1 and Ishikawa STING$^{-/-}$ cells.

Table S1. qPCR primers.

Primer name	sequences
Sting F	5′-CCAGAGCACACTCTCCGGTA-3′
Sting R	5′-CGCATTTGGGAGGGAGTAGTA-3′
cGAS F	5′-CACGAAGCCAAGACCTCCG-3′
cGAS R	5′-GTCGCACTTCAGTCTGAGCA-3′
IFNβ F	5′-ATGACCAACAAGTGTCTCCTCC-3′
IFNβ R	5′-GGAATCCAAGCAAGTTGTAGCTC-3′

ccl5 F	5′-CCAAGCAGTCGTCTTTGTCAC-3′
ccl5 R	5′-CTCTGGGTTGGCACACACTT-3′
CXCL10 F	5′-GTGGCATTCAAGGAGTACCTC-3′
CXCL10 R	5′-TGATGGCCTTCGATTCTGGATT-3′
HDAC1 F	5′-CTACTACGACGGGGATGTTGG-3′
HDAC1 R	5′-GAGTCATGCGGATTCGGTGAG-3′
HDAC3 F	5′-CCTGGCATTGACCCATAGCC-3′
HDAC3 R	5′-CTCTTGGTGAAGCCTTGCATA-3′
HDAC2 F	5′-ATGGCGTACAGTCAAGGAGG-3′
HDAC2 R	5′-TGCGGATTCTATGAGGCTTCA-3′
HDAC8 F	5′-TCGCTGGTCCCGGTTTATATC-3′
HDAC8 R	5′-TACTGGCCCGTTTGGGGAT-3′
GADPH F	5′-GGAGCGAGATCCCTCCAAAAT-3′
GADPH R	5′-GGCTGTTGTCATACTTCTCATGG-3′
